# A Market Basket Assessment: Prices and Availability of Healthy Foods Across SNAP-Authorized Food Outlets in Counties With High Obesity Rates in Mississippi

**DOI:** 10.5888/pcd18.210173

**Published:** 2021-12-02

**Authors:** Elizabeth Canales, Linlin Fan, David R. Buys, Marven D. Cantave

**Affiliations:** 1 Department of Agricultural Economics, Mississippi State University, Starkville, Mississippi; 2 Department of Agricultural Economics, Sociology and Education, Pennsylvania State University, State College, Pennsylvania; 3 Department of Food Science, Nutrition and Health Promotion, Mississippi State University, Starkville, Mississippi; 4 Mississippi State University Extension, Belzoni, Mississippi; 5 Now with Feeding America, Lexington, Mississippi

## Abstract

**Introduction:**

The Mississippi Delta is predominantly rural and ranks among the US regions with the highest obesity rates. Throughout the US, rural and low-income communities have limited access to healthy foods. Given the interrelation between the quality of the food environment and the healthfulness of diets and obesity rates, the food environment is an important public health concern in these communities.

**Methods:**

We conducted a retail assessment in July 2019 in the Delta region of Mississippi and evaluated prices and availability of healthy foods at Supplemental Nutrition Assistance Program–accepting retail establishments using the validated Market Basket Assessment Tool. We used regression analysis to identify differences in prices and availability of healthy foods across food retail formats.

**Results:**

The healthy foods availability and quality score for convenience stores, which comprise the highest proportion of store formats in the region, was 70% lower than for supermarkets. Compared with the prices at supermarkets, the prices at convenience stores were 48% higher for grains, 35% higher for fruit and vegetables, 73% higher for meats, and 95% higher for beans, seeds, and nuts. The healthfulness of foods available at dollar stores was also lower than the healthfulness at supermarkets, but prices were generally similar.

**Conclusion:**

The availability of supermarkets and grocery stores was limited in the study area, but the concentration of convenience stores was high. Overall, access and affordability of healthy foods were restricted in the counties studied; these findings are useful for intervention development.

**Summary**

**What is already known on this topic?**

Across the US, the prevalence of obesity is higher in nonmetropolitan areas than in metropolitan areas, which is partially explained by less access to healthy foods. Poor food environments have been linked to poor diets and obesity.

**What is added by this report?**

The availability of healthy foods at convenience stores — prevalent throughout the Mississippi Delta — is significantly lower and prices are significantly higher (35%−95%) than at supermarkets and other retail outlets.

**What are the implications for public health practice?**

Strategies aimed at increasing the supply of healthy foods at affordable prices at SNAP-authorized stores are needed to improve the dietary quality of consumers, especially for SNAP recipients.

## Introduction

Across the US, the prevalence of obesity is of concern to health professionals and policy makers, particularly as rates of obesity have increased throughout the country (1,2). Disparities in the prevalence of obesity are generally associated with socioeconomic conditions and environmental factors (3). One such factor is the food environment, which includes food access, and the availability and affordability of healthy foods (4,5). The composition of the food environment (ie, the type, variety, and prices of foods available) can influence a person’s purchasing and consumption choices (6–8).

Mississippi has a large rural population and is among the states with the highest obesity rates in the country (2). The Delta region of Mississippi has some of the greatest income inequality, highest rates of poverty, and highest prevalence of preventable nutrition-related chronic diseases in the US. An estimated 10% of the households in the state receive Supplemental Nutrition Assistance Program (SNAP) benefits and have limited access to grocery stores (6). Approximately 18% of the state population ― representing 83% of the population eligible to receive SNAP benefits ― receives SNAP benefits (6). Approximately 8% of all households in the study counties have no access to a car and are located more than 1 mile from a supermarket or large grocery store (6). As in many other US regions, disparities in the built environment, particularly in the food environment, may affect the quality of diets and, ultimately, health outcomes (7,8). Understanding these factors better will help identify local strategies and policies that promote food environments conducive to healthy eating.

Our study builds on earlier work conducted in the region (7,9) by analyzing the availability of healthy foods and comparing prices of healthy foods across food retail formats. This assessment of the food retail environment is part of a Centers for Disease Control and Prevention–funded grant with an overarching goal of increasing communities’ access to places offering healthy foods. The objectives of this research were to inform the implementation of initiatives that improve the healthfulness of the food system and to identify outreach, education, and intervention opportunities across food retail formats in the Mississippi Delta.

## Methods

We used the Market Basket Assessment Tool (MBAT) — a retail environment audit tool (10) — to evaluate the retail environment of the 8 counties with the highest obesity rates in the Delta region of Mississippi. The counties were Holmes, Humphreys, Issaquena, Leflore, Quitman, Sharkey, Sunflower, and Washington. We used the MBAT to collect information from 71 SNAP-authorized stores in July 2019. We audited 4 supermarkets, 17 medium-sized and small grocery stores, 14 dollar stores, and 36 convenience stores. This sample represents 100% of the supermarkets, 94% of grocery stores, 34% of dollar stores, and 19% of convenience stores that are SNAP-authorized in the study region. The convenience store category in this study included corner stores, gas stations, and pharmacies. The stores evaluated represent approximately 28% of the existing SNAP-authorized stores in the target region.

The stores studied were sampled from the list of US Department of Agriculture SNAP-authorized retailers in each county (11). We randomly selected stores for the audit. When randomly selected convenience stores were near each other, we audited only one of the stores, and we included a store in a different location. We used this approach to maximize geographic coverage. Because of the limited number of supermarkets and grocery stores and large number of convenience stores, we oversampled the former and undersampled the latter. Overall, approximately half of the stores audited were convenience stores.

### Survey instrument

The MBAT (10) is a 4-page tool used to record the availability, quality, and prices of food groups based on foods commonly consumed and the *2015–2020 Dietary Guidelines for Americans* (12). The MBAT is a practical and easy-to-use retail audit tool, particularly when the retail assessment focuses on the availability of healthy foods promoted for consumption based on the US dietary guidelines. This tool has been tested for interrater and test–retest reliability (10). The MBAT covers 6 food groups: grains, fruit, vegetables, meat, dairy and eggs, and dried beans, seed, nuts, and nut butters (Box). Two 2-member teams trained by the developer of the MBAT collected the data. They approached managers at each store and asked permission to collect the information. Except for 1 convenience store, managers at all stores allowed the teams to conduct the audit. Store managers were not interviewed for the assessment. Given that this study did not involve human subjects, it was exempt from review by the Institutional Review Board for the Protection of Human Subjects at Mississippi State University.

**Box. List of Food Groups and Food Products Audited (Availability, Quality, and Price) as Part of Market Basket Assessment Tool (MBAT), SNAP-Authorized Food Outlets in Counties With High Obesity Rates, Mississippi, 2019**

**Grains**

100% Whole-wheat or whole-grain bread, healthy cold cereal, hot cereal (oatmeal without added sugar, whole-grain cream of wheat, grits, other [fill in blank]), baked goods (whole-grain bagels, whole-grain English muffins, whole-grain tortillas, other [fill in blank]), other grains (brown rice, whole-grain pasta, unflavored or low-fat popcorn, other [fill in blank])

**Fruit (fresh, frozen, or canned)**

Apples, bananas, oranges, melons, peaches, pears, pineapple, berries, other [fill in blank]

**Vegetables (fresh, frozen, or canned)**

Asparagus, beans, beets, broccoli, cabbage, carrots, cauliflower, corn, cucumber, green beans, potatoes, spinach, tomatoes, other [fill in blank]

**Meat (fresh, frozen, or canned)**

Lean ground beef, chicken breast, chicken pieces, ground chicken, whole chicken, ground turkey, turkey breast, clams, flounder, tilapia, tuna, salmon, sardines, shrimp, other [fill in blank]; lunch meats (chicken breast, turkey breast, ham, other [fill in blank])

**Dairy and eggs**

Low-fat or fat-free milk, low-fat or fat-free cheese, low-fat or fat-free yogurt or Greek yogurt, eggs, egg mixtures/products, other [fill in blank]

**Dried beans, seeds, nuts, and nut butters**

Dried black beans, dried garbanzo beans/chickpeas, dried lentils, dried pinto beans, pumpkin seeds, sunflower seeds, almonds, cashews, mixed nuts, peanut butter, other [fill in blank]

Source: Market Basket Assessment Tool (10).

The price recorded for each product was the original price of the lowest per-unit (weight or volume) selling price available at the store, excluding promotions and discounts. We recorded information on expired products because we factored it into the calculation of the availability and quality score. When fat-free or low-fat milk, cheese, or yogurt were unavailable, the auditors recorded the price of the product with the lowest fat content.

### Analysis

**Healthy foods availability and quality score.** We calculated the number of items available in each food group and estimated a healthy food availability and quality score (hereinafter referred to as *healthy score*), which we then compared across food outlets. We constructed a score for each store according to the availability and quality of healthy products available in each food group (Box) using the MBAT scoring mechanism (Appendix). The maximum point score for each food group was as follows: grains, 5; fruit, 10; vegetables, 10; meat, 4; dairy and eggs, 5; and dried beans, seeds, nuts, and nut butters, 6. The total maximum point score is 40, with higher scores indicating greater availability of healthy foods, greater variety, and better quality. We used a Kruskal–Wallis test to assess statistical differences in the scores of each food group across store formats. For the total score, we estimated a linear regression on the log-transformed score (dependent variable) to evaluate the differences in healthy scores across food retail formats. In this regression, the explanatory variables were indicator variables for store format, and supermarket was used as the referent.

**Food prices.** We used linear regression to evaluate differences in food prices across store formats, where the dependent variable was the log-transformed food price (in dollars per ounce). In addition to store format indicators, we included indicator variables for food groups to control for price difference across main groups. For the food group indicators, we combined fruit and vegetables into 1 category. Because stores could have different pricing structures for different food items, we included interaction effects between store format and food groups. Although our target region was largely rural, some cities have a larger population and wider access to stores and supermarkets. Thus, we included city-level dummy variables to control for potential price differences resulting from spatial heterogeneity. As robustness checks, we estimated several alternative specifications: 1) no random effects or clustered SEs, 2) clustered SEs at the subgroup level to allow for within-subgroup food category correlation, 3) clustered SEs at the store level to allow for within-store correlation, 4) food subgroup–level random effects, and 5) store-level random effects. We used the results from Model 5 (store-level random-effects regression) to calculate the average marginal effect of store format on prices across food groups. The magnitude and significance of the marginal effects across the 5 models (not reported because of space limitations) were similar. We used Stata version 17 (StataCorp LLC) to estimate the linear regressions and marginal effects. We reported significance at the 1% (*P* < .01), 5% (*P* < .05), and 10% (*P* < .10) levels.

## Results

Of the 8 counties evaluated, 3 counties (Leflore, Sunflower, and Washington) had a supermarket (Walmart and/or Kroger). One county (Issaquena) had no grocery store, and we could only identify 1 small convenience store in that county. The remaining counties had a small number of grocery stores (generally Piggly Wiggly, SuperValu Foods, or small local grocery stores). The predominant food retail format in all counties was convenience stores.

### Healthy foods availability and quality score

**Figure F1:**
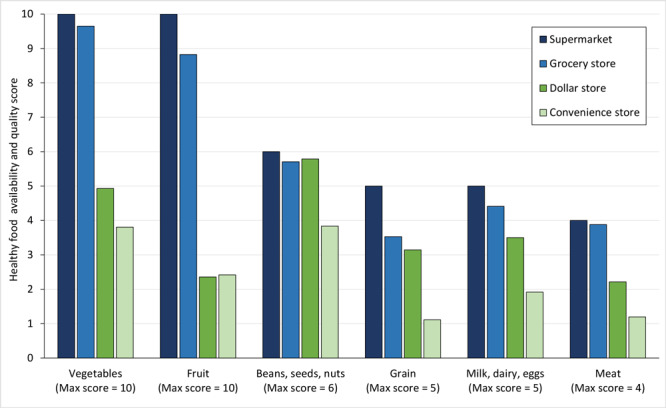
Average healthy food availability and quality score for each food group based on the Market Basket Assessment Tool in 8 counties in the Mississippi Delta region, 2019.

Supermarkets provided the healthiest assortment of foods, followed by grocery stores [Fig F1](Figure). For all food groups, the Kruskal–Wallis test indicated significant differences in scores across store formats (all *P *values <.01). We found a gap between the scores for convenience stores and scores for supermarkets and grocery stores, and the difference was most striking for fruit and vegetables (Figure). This gap is explained by the lack of fruit and vegetable options. For example, none of the convenience stores carried frozen fruit, and only a few carried fresh fruit and vegetables (predominantly bananas, oranges, cabbage, potatoes, or tomatoes). Convenience stores and dollar stores generally sold eggs and milk — albeit in smaller packages than at supermarkets or grocery stores — but low-fat cheese and yogurt were rarely available. 

**Table:** 

Store Format	Vegetables (Max score = 10)	Fruit (Max score = 10)	Beans, seeds, nuts (Max score = 6)	Grain (Max score = 5)	Milk, dairy, eggs (Max score = 5)	Meat (Max score = 4)
Supermarket	10.0	10.0	6.0	5.0	5.0	4.0
Grocery store	9.6	8.8	5.7	3.5	4.4	3.9
Dollar store	4.9	2.4	5.8	3.1	3.5	2.2
Convenience store	3.8	2.4	3.8	1.1	1.9	1.2

Convenience stores did not offer meat products beyond lunch meats. These lunch meats often did not satisfy the sodium-limit requirement (<360 mg sodium per serving) to be considered healthy and were therefore marked as unavailable. For the beans, seeds, and nuts group, we found a smaller gap in availability between convenience stores and other retail formats. Convenience stores were more likely to carry foods with longer shelf life (eg, dried beans) and snack-sized seed and nut packages. Similarly, although convenience stores did not carry whole-grain breads, baked goods, and pastas, they consistently carried oats and grits.

In the regression on the log-transformed total score as a function of food retail format, overall, we found grocery stores to be as healthy as supermarkets. That is, the average healthy score of grocery stores was not significantly different from that of supermarkets (*P *value >.10). Compared with the average healthy foods availability score for supermarkets, the score for dollar stores was 45.6% (95% CI, −69.5% to –3.0%) lower and the score for convenience stores was 70.2% (95% CI, −82.6% to −49.1%) lower.

### Difference in prices across food retail formats

Overall, we found significant differences in healthy food prices across store formats (Table 1). However, these price differences were not consistent across food groups. Calculation of the average marginal effect of store format on prices across food groups showed that, relative to prices at supermarkets, prices at convenience stores were 35.4% (95% CI, 16.7%−57.2%) higher for fruit and vegetables, 73.5% (95% CI, 49.2%−101.7%) higher for healthy meats, 47.9% (95% CI, 5.5%−107.3%) higher for healthy grains, and 95.3% (95% CI, 57.5%−142.1%) higher for healthy beans, seeds, and nuts (Table 2). The prices of milk and eggs were not significantly different, possibly because of the small number of observations in this category. Except for beans, seeds, and nuts, the prices of food products at dollar stores were not significantly higher than prices at supermarkets. Relative to supermarkets, the prices of fruit and vegetables at dollar stores were found to be 25.8% lower (95% CI, −33.4% to −17.2%), driven primarily by low prices of frozen and canned products. Although the prices at dollar stores might be similar to prices at supermarkets, these stores carry a small inventory of products, especially fresh products, and as a result had a lower healthy score (Figure). The overall prices and availability of healthy foods at grocery stores were similar to those at supermarkets.

**Table 1:** Regression Results of the Log of Healthy Food Prices^a^ as a Function of Store Format, SNAP-Authorized Food Outlets in Counties With High Obesity Rates, Mississippi, 2019

Variable	Model 1^b^	Model 2^c^	Model 3^d^	Model 4^e^	Model 5^f^
**Constant**	−1.976 (0.302)^g^	−1.976 (0.714)^h^	−1.976 (0.086)^g^	−1.976 (0.714)^g^	−1.985 (0.086)^g^
**Store format (reference: supermarket)**					
Grocery store	0.159 (0.120)	0.159 (0.037)	0.159 (0.097)^g^	0.159 (0.037)^g^	0.165 (0.098)^i^
Dollar store	0.351 (0.128)^g^	0.351 (0.170)^i^	0.351 (0.083)^g^	0.351 (0.170)^h^	0.360 (0.084)^g^
Convenience store	0.666 (0.121)^g^	0.666 (0.153)^g^	0.666 (0.109)^g^	0.666 (0.153)^g^	0.669 (0.110)^g^
**Food groups (reference: beans, seeds, nuts)**					
Dairy and eggs	−0.742 (0.184)^g^	−0.742 (0.452)	−0.742 (0.042)^g^	−0.742 (0.452)	−0.741 (0.042)^g^
Fruit and vegetables	−0.593 (0.110)^g^	−0.593 (0.485)	−0.593 (0.042)^g^	−0.593 (0.485)	−0.593 (0.043)^g^
Grains	−0.555 (0.148)^g^	−0.555 (0.544)	−0.555 (0.090)^g^	−0.555 (0.544)	−0.557 (0.089)^g^
Meats	0.288 (0.121)^h^	0.288 (0.481)	0.288 (0.050)^g^	0.288 (0.481)	0.291 (0.050)^g^
**Store format and food group interactions**					
Grocery store × dairy and eggs	−0.214 (0.209)	−0.214 (0.132)	−0.214 (0.080)^g^	−0.214 (0.132)	−0.214 (0.080)^g^
Grocery store × fruit and vegetables	−0.159 (0.128)	−0.159 (0.070)^h^	−0.159 (0.076)^h^	−0.159 (0.070)^h^	−0.159 (0.077)^h^
Grocery store × grains	−0.021 (0.173)	−0.021 (0.121)	−0.021 (0.137)	−0.021 (0.121)	−0.021 (0.137)
Grocery store × meats	−0.194 (0.142)	−0.194 (0.114)	−0.194 (0.074)	−0.194 (0.114)	−0.197 (0.074)
Dollar store × dairy and eggs	−0.474 (0.225)^h^	−0.474 (0.183)^h^	−0.474 (0.081)^g^	−0.474 (0.183)^g^	−0.473 (0.081)^g^
Dollar store × fruit and vegetables	−0.658 (0.137)^g^	−0.658 (0.179)^g^	−0.658 (0.061)^g^	−0.658 (0.179)^g^	−0.658 (0.061)^g^
Dollar store × grains	−0.181 (0.184)	−0.181 (0.291)	−0.181 (0.124)	−0.181 (0.291)	−0.179 (0.124)
Dollar store × meats	−0.273 (0.154)	−0.273 (0.258)	−0.273 (0.067)^g^	−0.273 (0.258)	−0.275 (0.066)^g^
Convenience store × dairy and eggs	−0.606 (0.218)^g^	−0.606 (0.174)^g^	−0.606 (0.135)^g^	−0.606 (0.174)^g^	−0.608 (0.134)^g^
Convenience store × fruit and vegetables	−0.377 (0.136)^g^	−0.377 (0.210)^i^	−0.377 (0.104)^g^	−0.377 (0.210)^g^	−0.366 (0.104)^g^
Convenience store × grains	−0.288 (0.188)	−0.288 (0.244)	−0.288 (0.154)	−0.288 (0.244)	−0.278 (0.154)
Convenience store × meats	−0.118 (0.156)	−0.118 (0.223)	−0.118 (0.095)	−0.118 (0.223)	−0.118 (0.095)

Abbreviation: SNAP, Supplemental Nutrition Assistance Program.

a Dependent variable is log of food price, in dollars per ounce. Data were collected via store audit in July 2019. Only healthy foods within each food group were included in the audit. Data presented are regression coefficients (SE).

b Model 1: Included city-level dummy variables to control for potential price differences resulting from spatial heterogeneity. No random effects. No clustered SEs. No. of observations: 2,636. *R* ^2^ = 0.334.

c Model 2: Included city-level dummy variables to control for potential price differences resulting from spatial heterogeneity. No random effects. Clustered SEs: food subgroup. The following 16 subgroup food categories were included: fruit and vegetables (fresh, frozen, and canned), meats (fresh meat, frozen meat, canned meat, fresh seafood, frozen seafood, canned seafood, lunchmeat), grains (cereal and grains, baked goods), beans, seeds, nuts (beans, nuts), dairy and eggs (milk and dairy, eggs). No. of observations: 2,636. *R* ^2^ = 0.334.

d Model 3: Included city-level dummy variables to control for potential price differences resulting from spatial heterogeneity. No random effects. Clustered SEs: store level. No. of observations: 2,636. *R* ^2^ = 0.334.

e Model 4: Included city-level dummy variables to control for potential price differences resulting from spatial heterogeneity. Random effects: food subgroup. Clustered SEs: food subgroup. Clustered errors are equivalent to robust SEs based on the variable associated with the random effects. No. of observations: 2,636. *R* ^2^ = 0.335.

f Model 5: Included city-level dummy variables. Random effects: store level. Clustered SEs: store level. Clustered errors are equivalent to robust SEs based on the variable associated with the random effects. No. of observations: 2,636. *R* ^2^ = 0.335.

g *P* < .01.

h *P* < .05.

i *P* < .10.

**Table 2:** Marginal Effects of Store Format, Relative to Supermarkets, on Food Prices Across Food Groups, SNAP-Authorized Food Outlets in Counties With High Obesity Rates, Mississippi, 2019

Store Format	Marginal Effect^a^ (SE) [*P* Value]^b^	Marginal Effect, % (95% CI)^c^
**Grocery store**		
Beans, seeds, nuts	0.165 (0.098) [.09]	18.0 (−2.6 to 42.9)
Dairy and eggs	−0.048 (0.082) [.56]	−4.7 (−18.9 to 12.0)
Fruit and vegetables	0.006 (0.049) [.90]	0.6 (−8.6 to 10.8)
Grains	0.144 (0.155) [.35]	15.5 (−14.8 to 56.6)
Meats	−0.031 (0.079) [.69]	−3.1 (−17.1 to 13.2)
**Dollar store**		
Beans, seeds, nuts	0.360 (0.084) [<.001]	43.4 (21.6 to 69.1)
Dairy and eggs	−0.113 (0.086) [.19]	−10.7 (−24.5 to 5.7)
Fruit and vegetables	−0.298 (0.056) [<.001]	−25.8 (−33.4 to −17.2)
Grains	0.181 (0.146) [.21]	19.9 (−9.9 to 59.5)
Meats	0.085 (0.077) [.27]	8.9 (−6.4 to 26.6)
**Convenience store**		
Beans, seeds, nuts	0.669 (0.110) [<.001]	95.3 (57.5 to 142.1)
Dairy and eggs	0.062 (0.112) [.58]	6.4 (−14.7 to 32.6)
Fruit and vegetables	0.303 (0.076) [<.001]	35.4 (16.7 to 57.2)
Grains	0.391 (0.172) [.02]	47.9 (5.5 to 107.3)
Meats	0.551 (0.077) [<.001]	73.5 (49.2 to 101.7)

Abbreviation: SNAP, Supplemental Nutrition Assistance Program.

a Average marginal effects of store format, relative to supermarkets, on the log of food prices across food groups were estimated using the results from Model 5 in Table 1. Data were collected via store audit in July 2019.

b Percentage difference was calculated as *e* ^ME^ − 1.

c Calculated by *t* test.

## Discussion

Although the intake of energy (calories) from diets in the Mississippi Delta region has not been found to diverge significantly from that of the rest of the US population, the reported intake of protein, dairy products, fruit, and vegetables is significantly lower (8). Limited access to high-quality foods in this region may explain the inadequate intake of important nutrients, particularly among African Americans (8). Previous studies found that small and medium-sized food stores in the lower Mississippi Delta carried only 50% of the foods in the Thrifty Food Plan (https://www.fns.usda.gov/cnpp/usda-food-plans-cost-food-reports), with convenience stores carrying only 28% (7). However, to our knowledge, no studies since Connell et al (7) have sought to explore the make-up of food outlets in this region.

Results from our assessment indicate limited access to supermarkets and grocery stores but broader access to convenience stores, which have an inadequate inventory of healthy foods. Convenience stores consistently lack fresh and frozen fruit, vegetables, and meat products. However, although limited in variety and inventory, convenience stores generally offer canned fruit and vegetables. Our findings also indicate that prices of healthy foods were consistently higher (35%−95%) at convenience stores than at supermarkets even after controlling for price variability caused by store heterogeneity and location effects. This finding is consistent with those of Fan et al (9) in a study that used scanner data and found that prices of healthy food were consistently higher in counties with high obesity rates in Mississippi compared with the rest of the state.

Residents of counties with limited access to supermarkets and grocery stores in the Delta region of Mississippi face limited access to healthy foods. Similar insights have been found on the healthfulness of diets and access to supermarkets in national studies (13) and studies focusing on underserved communities (ie, communities that include members of minority populations or individuals who have experienced health disparities) (14). Insights from our study also indicate that when healthy products are available at neighborhood convenience stores, prices are significantly higher than prices at supermarkets. Based on observations during our retail audit, we contend that for some products, the quality found in convenience stores is also likely to be lower than the quality found in other store formats. Similarly, the healthfulness of products at dollar stores — another store format prevalent throughout the region — was significantly lower than the healthfulness of products at supermarkets. However, when healthy foods were available at dollar stores, the prices of healthy foods were generally similar to the prices at supermarkets, making these store formats a better alternative than convenience stores.

The problem of insufficient availability of healthy foods locally, particularly of fresh fruit and vegetables, is aggravated by limited access to transportation ― both of personal vehicles and public transportation ― in this region (6,15). Results from a 2020 community-based survey in the same target counties (16) revealed that residents traveled on average 13 miles to the nearest full-service grocery store and 25% of residents traveled more than 20 miles. This community-based survey (16) also showed that 72% of the respondents purchased foods at dollar stores and 37% purchased foods at convenience stores. As the data from the community-based survey indicated, residents in the Mississippi Delta generally travel long distances to access full-service food stores, and as a result, residents rely on dollar stores and convenience stores for some of their food purchases.

Higher obesity rates in nonmetropolitan rural areas than in metropolitan areas could be partially explained by less access to healthy foods (3,17). Limitations in food access might be aggravated by the high food prices in many low-income and rural areas, hindering the affordability of a healthy diet (18). Places with poor food access, as measured by the distance residents must travel to the nearest supermarket, also have fewer healthy foods available ─ particularly fewer fruit and vegetable options (13). Poor food environments have also been linked to lower dietary quality and higher rates of obesity among residents in affected areas (4).

Our results and understanding of the healthfulness and price differences across food retail formats in this region have important implications because they affect a considerable segment of the population, specifically those who acquire foods at retail outlets other than supermarkets and grocery stores. In our target counties, approximately 36% of the population have low levels of access to supermarkets or grocery stores (eg, live more than 1 mile away from a supermarket if in an urban area, or more than 10 miles away if in a rural area), 24% of households are low income and have low levels of store access, and 10% receive SNAP benefits and have low levels of store access (6). Therefore, many residents resort to convenience or dollar stores to meet their food needs.

Poor food environments, such as those analyzed in our study, can directly affect dietary quality. Direct links may exist among store access, healthy foods assortment, and consumption of healthy foods (5,13). For example, consumers who do not purchase most of their food at supermarkets have been found to consume less fruit and vegetables than people who purchase most of their food at supermarkets (19). Other studies have found an association between poor food environments and high rates of obesity (4). High food prices also have ramifications for food insecurity. One study (20) found that SNAP recipients in regions with high food prices were more likely to experience food insecurity than recipients in regions where food prices were lower. Thus, SNAP recipients in the Mississippi Delta ― a region with limited access to supermarkets and a high concentration of convenience stores offering limited and high-priced healthy foods ― are potentially more susceptible to food insecurity than if they lived in an area with greater access to supermarkets and affordable healthy foods.

Given the extent of the obesity problem and poor health outcomes in the target region, the deficiency of the food environment — characterized by limited affordability and access to healthy foods — calls for multisector strategic action. Strategies aimed at increasing the supply of healthy foods at affordable prices can improve dietary quality (21). First, given that convenience stores are prominent throughout the region, the feasibility of healthy corner stores initiatives could be explored (22). Because convenience stores mainly carry shelf-stable products, efforts could begin by targeting improvements in the assortment of longer shelf-life healthy foods (eg, canned fruit and vegetables). Increasing the healthfulness of the food assortment at existing SNAP-accepting stores could contribute to the program goal of ensuring access to nutritious food within the budgetary limitations of program participants (23). Second, given that the assortment of foods in a particular area is the result of an interaction of supply and demand (24), interventions could also target the demand side by incorporating marketing, promotion, and educational efforts (17,22,25). Promotion initiatives could include efforts to make healthy foods more affordable via subsidies (26). Such efforts have increased the consumption of healthy foods such as fruit and vegetables (27). Third, the interacting roles of food access, nutrition, health-related outcomes, and associated economic impacts could be considered. With sufficient evidence to connect food access to local, state, and regional economic viability, local governments could consider tax incentives or tax increment financing to attract and keep reasonably sized food outlets that offer suitable food for positive health outcomes (28). However, these strategies may only have a marginal effect and the long-term economic viability of these stores may preclude the long-term feasibility of these strategies (26).

Our study has several limitations. First, because we collected and evaluated data on the lowest listed price for products available at each store, we did not compare products of the same quality (eg, same brand). In some product categories, the cheapest product recorded at a convenience store may have been of lower quality than the lowest priced product at a supermarket. Thus, our results provide insights into the availability of healthy foods and the lowest price at which they can be found across retail formats. Second, the MBAT, which is a simplified audit tool compared with other tools such as the Nutrition Environment Measures Survey, reduced the burden of the audit and provided sufficient information to assess the availability of healthy foods in the counties examined. However, we did not evaluate the availability and prices of beverages and unhealthy foods. Third, we did not explore the spatial distribution of stores in the area and the sociodemographic profile of the neighborhoods in the target counties. We included city-level dummy variables in our price regression to control for spatial variability; however, other sources of spatial correlation between stores may exist.

### Conclusion

Many communities across the Mississippi Delta have greater access to convenience stores than to supermarkets and grocery stores. Convenience stores lack an adequate supply of fresh and frozen fruit, vegetables, and meat products but do often offer canned fruit and vegetables. Healthy foods are consistently more expensive (35%−95%) at convenience stores. These findings, which demonstrate a lack of access to healthy food, offer an additional possible explanation for the poor health outcomes often associated with residents in the Mississippi Delta. Suggested strategies to reverse these disparities in access to healthy food include healthy corner stores initiatives, marketing and educational efforts about the importance of healthy food choices, SNAP subsidies to purchase fruit and vegetables, and support of stores that offer a balanced range of foods.

Understanding the food environment is important because it is associated with household food choices and the ability of consumers to access and afford healthy diets. Insights from our results and discussion of the differences in the availability and prices of different food groups across various food retail formats may extend to other regions in the US ― particularly rural areas and low-income and racial and ethnic minority neighborhoods ― where convenience stores generally comprise the highest proportion of stores available (29).

## Appendix. Text of Market Basket Assessment Tools Score Sheet^a^

**Table:**

Category	Availability	Quality	Max Possible Score
Grains	Whole-grain breads = 1 point Healthy cold cereal available = 1 point Whole-grain hot cereal available = 1 point Whole-grain baked goods available = 1 point Other whole-grain available = 1 point	Not applicable	5
Fruit	<5 varieties = 1 point 5–9 varieties = 2 points ≥10 varieties = 3 points Fresh fruit available = 2 points Canned fruit available = 1 point Frozen fruit available = 1 point	Fresh: 25%–50% acceptable = 1 point 50%–75% acceptable = 2 points ≥75% acceptable = 3 points	10
Vegetables	<5 varieties = 1 point 5–9 varieties = 2 points ≥10 varieties = 3 points Fresh vegetable available = 2 points Canned vegetable available = 1 point Frozen vegetable available = 1 point	Fresh: 25%–50% acceptable = 1 point 50%–75% acceptable = 2 points ≥75% acceptable = 3 points	10
Meat	Fresh meat available = 1 point Frozen meat available = 1 point Canned meat available = 1 point Lunch meat available = 1 point	Expired products available = −1 point	4
Dairy and eggs	Low-fat or fat-free milk available = 1 point Low-fat or fat-free cheese available = 1 point Low-fat or fat-free yogurt available = 1 point Eggs or egg products available = 2 points	Expired products available = −1 point	5
Dried beans, seeds, nuts, and nut butters	2-3 varieties = 1 point >3 varieties = 2 points Dried beans available = 1 point Seeds available = 1 point Nuts available = 1 point Nut butters available = 1 point	Not applicable	6
Up to 40 points possible (Availability + Quality)			Total =

^a^ Source: Reproduced from Misyak et al (10). The total healthy score for a store is calculated as the sum of points from all food groups for both quality and availability; maximum score, 40 points.
